# Physical activity and joint health: Implications for knee osteoarthritis disease pathophysiology and mechanics

**DOI:** 10.1113/EP092240

**Published:** 2024-12-13

**Authors:** Karl Morgan, Joshua Carter, Dario Cazzola, Jean‐Philippe Walhin

**Affiliations:** ^1^ Department for Health University of Bath Bath UK; ^2^ Centre for Health and Injury and Illness Prevention in Sport (CHI^2^PS), Department for Health University of Bath Bath UK; ^3^ Centre for Nutrition and Exercise Metabolism (CNEM), Department for Health University of Bath Bath UK; ^4^ Centre for Sport, Exercise and Osteoarthritis Versus Arthritis University of Bath Bath UK; ^5^ Centre for the Analysis of Motion, Entertainment Research and Applications (CAMERA) University of Bath Bath UK

**Keywords:** biomechanics, human physiology, knee, mechanotransduction, osteoarthritis, physical activity

## Abstract

Knee osteoarthritis is experienced by hundreds of millions of people worldwide and is a major cause of disability. Although enhancing physical activity levels and the participation in exercise programmes has been proved to improve the debilitating illness of osteoarthritis, many do not engage in recommended levels of physical activity. One of the reported barriers to exercise engagement is the perception that physical activity can damage joint health and is attributed to the incorrect perception of ‘wear and tear’. We posit that these perceptions arise from uncertainty and ambiguity generated from conflicting research findings. In this review, we explore the complex relationship between knee osteoarthritis and physical activity. We demonstrate how factors contribute to the uncertainty around the effects of physical activity on joint tissue metabolism, structure and function. The aim of this review is to demonstrate how a nuanced approach to the relationship between physical activity and knee osteoarthritis can help to dispel misconceptions, leading to better management strategies and improved quality of life for patients.

## Abstract (highlights)

- **What is the topic of this review?**
  Although physical activity reduces knee pain, improves function and enhances quality of life, many people with knee osteoarthritis believe that physical activity will cause joint damage. In this review, we examine the existing evidence on how physical activity influences the pathophysiology of knee osteoarthritis disease.
- **What advances does it highlight?**
  Contrasting study outcomes might be a contributing factor to the perception that exercise is harmful to the affected joint in individuals with knee osteoarthritis. We demonstrate how a nuanced approach to the relationship between physical activity and knee osteoarthritis can help to dispel misconceptions and guide future research.

## 1 INTRODUCTION

Osteoarthritis (OA) is a highly prevalent condition, affecting 15% of the global adult population, which translates to >500 million people (Vos et al., 2015). The knee is the joint most commonly affected by OA (Long et al., 2022), which poses serious medical and societal concerns. It is associated with a high financial burden (Leifer et al., 2022), reduced quality of life (Picavet & Hoeymans, 2004), a high prevalence of comorbidities (Kadam et al., 2004) and a higher risk of all‐cause mortality (Cleveland et al., 2019). Although there is currently no cure for knee OA, treatments such as exercise and weight loss can mitigate the often‐debilitating symptoms of the condition (Fransen et al., 2015). However, there is a concerning perception that physical activity is harmful or damaging to the affected joint in this population. In fact, 69% of people with osteoarthritic knee pain hold the unconscious belief that exercise is more dangerous for them than for the average person without pain (Pulling et al., 2024). This perception aligns with the persistent narrative that knee OA is a ‘wear and tear’ arthritis, whereby exercise results in anatomical damage, a conception that remains prevalent in medical, media and patient representations. However, this view oversimplifies the complex aetiology of the disease.

A partial explanation for this misconception is the lack of clear information from human studies on the impact of exercise on OA disease pathogenesis. Consequently, the aim of this narrative review is to elucidate the complex relationship between knee OA and physical activity. Specifically, in this review, we will: (1) examine the existing evidence on how physical activity influences the pathophysiology and progression of knee OA; (2) identify and analyse the factors contributing to the misconception that exercise is harmful to individuals with knee OA; and (3) highlight the gaps in current research and propose directions for future studies to provide clearer guidance for both clinicians and patients. By addressing these objectives, this review demonstrates how a nuanced approach to the relationship between physical activity and knee OA can help to dispel misconceptions, leading to better management strategies and improved quality of life for patients.

## 2 WHAT IS KNEE OSTEOARTHRITIS?

Knee osteoarthritis is best defined by the Osteoarthritis Research Society International (Kraus et al., 2015). The society states that OA is a joint disorder characterized by cell stress and extracellular matrix degradation (a key component of the cartilage), which is initiated by micro‐ and macro‐injury. Sustaining the micro‐ and macro‐injury activates maladaptive repair responses, including pro‐inflammatory pathways of innate immunity. This results in the disease manifesting initially as a molecular derangement (e.g., abnormal joint tissue metabolism), followed by anatomical and/or physiological derangements (characterized by cartilage degradation, bone remodelling, osteophyte formation, joint inflammation and/or loss of normal joint function). The initiation of the disease processes then increases the likelihood of experiencing illness (e.g., pain, stiffness, swelling and crepitus).

### 2.1 Disease

Knee OA disease involves pathophysiological degradation of joint tissues, including the hyaline cartilage, bone, synovium, ligament, periarticular fat, meniscus and muscle (Katz et al., 2021). These pathological changes are driven by a metabolic imbalance of the tissue towards a catabolic state, which manifests as a reduction in tissue size and altered composition. Disease activity is often quantified using imaging modalities to measure the size or infer the composition of the tissue of interest. Various imaging modalities are used to visualize joint structure, including radiography, magnetic resonance imaging and ultrasonography (Hunter et al., 2015). Furthermore, synovial, serum or plasma, and urinary biomarkers are used to determine disease activity by measuring protein quantities and metabolites of key structural proteins (fragments of synthesis or degradation). Resting biomarker concentrations provide an indication of the physiological milieu of the joint and can reflect an altered metabolic state within a joint preceding structural deterioration in a relatively non‐invasive manner. A biomarker group of interest has been outlined (Kraus et al., 2017); nevertheless, research is yet to determine definitively a single or group of serum/urinary biomarkers capable of distinguishing OA joints from healthy joints or predicting joint disease.

### 2.2 Illness

Illness refers to symptoms of knee OA such as pain (often called the cardinal symptom), stiffness, joint instability, swelling, crepitus and reduced function (Hunter et al., 2008). It is the illness that drives people to seek medical care, given the impact it has on the ability to perform daily tasks. Illness is measured using symptom reporting through questionnaires and clinical assessment (Collins et al., 2011; Hunter & Bierma‐Zeinstra, 2019). Clinical features of OA in the knee joint include functional limitation, bony enlargement, fixed flexion deformity, reduced range of flexion, swelling, deformity, effusion, instability and misalignment, crepitus (crunching or clicking) and reduced muscle strength (Kraus et al., 2015; Peat et al., 2012). Despite there being a poor correlation between knee OA disease and illness, experiencing the disease increases risk of illness (Hunter et al., 2008).

## 3 HOW IS IT DIAGNOSED?

Clinically, knee OA is primarily diagnosed through symptom reporting and physical examination. Radiography is used only when the diagnosis is uncertain or to rule out other conditions. The European League Against Rheumatism (EULAR) diagnostic criteria, using a combination of three symptoms (persistent knee pain, limited morning stiffness and reduced function) and three clinical signs (crepitus, restricted movement and bony enlargement), indicates that there is a 99% of chance of diagnosing radiographic knee OA (Zhang et al., 2010). Therefore, symptom evaluation and clinical assessment remain the standard for diagnosis, offering a practical solution for health‐care providers given the high costs of medical imaging and the burden it places on health‐care systems. However, in research contexts investigating disease activity, diagnosis of knee OA requires quantification of the joint anatomy, structure and composition, given the need to measure the pathophysiological degeneration of the joint tissues in a predisease or disease state. Using illness measurements in this context is not suitable, given the lack of specificity to the disease state of the tissue and the tissue. Modalities and classification systems to diagnose knee OA include radiography and semi‐quantitative grading scales (Hunter et al., 2015).

## 4 NO CURE, BUT HOW CAN KNEE OSTEOARTHRITIS BE TREATED?

Exercise is a cornerstone of care for people with knee OA (Katz et al., 2021). A meta‐analysis of 54 randomized control trials with an overall high quality has demonstrated that exercise can significantly ameliorate knee OA illness by reducing pain, improving physical function and enhancing quality of life (Fransen et al., 2015). Furthermore, considering the unequivocal association between adiposity and physical activity (Myers et al., 2017), exercise is often prescribed for body mass maintenance or reduction. Obesity causing higher internal mechanical load on the joint tissues is often attributed to contributing to knee OA symptomology (Katz et al., 2021). However, the role of mechanical loading (quantified by external measurements, such as joint moments) in the genesis or exacerbation of knee pain has been questioned, with body mass index discovered to be the mediating variable (Hutchison et al., 2023).

Despite the clear benefits of engagement in physical activity on reducing knee OA illness, <10% of people experiencing knee OA adhere to activity guidelines (White et al., 2013). Those who are experiencing knee OA report an aversion to joint‐loading exercise owing to the belief that exercise could further damage their vulnerable joint (Bunzli et al., 2019). Furthermore, those with knee OA might hold the unconscious belief that exercise and movement is dangerous for their joint (Pulling et al., 2024). Indeed, 69% of people with OA knee pain reported stronger implicit beliefs that exercise is more dangerous for those with OA in comparison to people without knee pain. We suggest that the heightened implicit feelings of danger during exercise in people with knee OA can be partly a response to a lack of clear evidence on the influence of physical activity on joint tissue health. These beliefs and implicit feelings coincide with ambiguous information, lack of information or conflicting information from clinicians regarding physical activity, which is a barrier to participation (Kanavaki et al., 2017). Consequently, understanding the physiological and mechanical responses to physical activity is of great interest to patients and clinicians alike, given the potential impact that knowledge would have on prevention of knee OA disease and therapeutic care for those experiencing the disease.

## 5 LESSONS FROM IN VITRO, EX VIVO AND ANIMAL MODELS

The effects of physical activity on knee OA disease pathogenesis can be inferred from in vitro and ex vivo research replicating the known physiological effects of activity; mechanical loading of the joint and anti‐inflammatory cytokines. Compelling evidence exists supporting the notion that physiological magnitudes of axial mechanical loading stimulate extracellular matrix synthesis and suppress pro‐inflammatory driven catabolism (Sanchez‐Adams et al., 2014). In the articular cartilage, this is driven by the metabolically active chondrocytes, which transduce the mechanical perturbations into biological signals (Vincent & Wann, 2019). Additionally, there is evidence linking adipokines to elevated systemic inflammation, which, in turn, elevates local joint inflammation and activates potent degraders of type II collagen and aggrecan (Berenbaum, 2013; Koskinen et al., 2011). Compression of cartilage can also attenuate the effect of local joint pro‐inflammatory‐driven cartilage degradation, considering that exposure of bovine cartilage explants to dynamic cyclical compression in a pro‐inflammatory milieu attenuated cartilage loss (Engstrøm et al., 2022). This might suggest that even in situations where chronic local knee joint pro‐inflammatory conditions exist (e.g., trauma, obesity), compression and the subsequent mechanotransduction might be protective of joint health. Although providing valuable insight into the pathophysiology of OA, these methods of loading joint tissues ex vivo are typically restricted to axial force‐driven perturbations. This limits the translatability of these discoveries, considering that in vivo, shear forces (anterior–posterior and mediolateral) are transferred across the joint tissues during ambulation. Future implementation of novel bioreactors (Lazaro‐Pacheco et al., 2024) that possess the ability to replicate loading protocols represented in vivo will help to improve our understanding of knee OA mechanobiology.

In addition to in vitro and ex vivo studies of the effects of physical activity on knee OA, animal models provide a level of control of physical activity that would perhaps not be feasible in humans. Studies suggest that a curvilinear relationship is present, whereby animals that engage in moderate amounts of daily physical activity have less tissue degradation than animals in the low and high daily activity groups (Bricca et al., 2017). Despite providing ecologically valid exposure of force transfer across joints, we must, however, interpret these results from animal models with caution, considering the limited generalizability from animal models (Hackam & Redelmeier, 2006).

Together, inferences from in vitro research and animal models suggest that there is an optimal zone, whereby too little or too much activity can lead to joint tissue catabolism; however, a moderate level will promote low adiposity, attenuate catabolism and, potentially, induce anabolism.

## 6 CONTRADICTIONS BETWEEN HUMAN STUDIES

There are several contrasting findings that make interpretation of the effects of physical activity on the risk of developing knee OA and on those already experiencing the condition difficult to ascertain.

### 6.1 Without knee osteoarthritis

A systematic review of randomized control trials and observational studies in middle‐aged to elderly people without OA discovered conflicting findings (Xu et al., 2023). One study found that higher physical activity levels were significantly associated with greater loss or lower current cartilage volume or thickness, whereas three other studies found protective effects of physical activity on cartilage volume. Contrastingly, five other studies did not find an association between physical activity and cartilage size. Of concern are results indicating that 40% of studies in the review discovered positive associations between physical activity level and cartilage defects. Furthermore, two of three studies demonstrated a positive association between physical activity and T2 relaxation times, whereas one demonstrated no association, which might suggest that physical activity has negative connotations on joint tissue health (Xu et al., 2023). Further research indicating a negative effect on joint tissue health is provided by a cohort study discovering that a higher weight‐bearing physical activity level was associated with increased odds of future incidence of radiographic knee OA, but low‐weight‐bearing activities were not (Wu et al., 2024). Of note is that when stratifying the population by lower‐body lean mass index, the increased odds were present only in the tertile consisting of 431 participants with the lowest lower‐body lean mass index, but not among patients in the middle or high tertile (Wu et al., 2024). Conversely, a study using meta‐analysis techniques investigating the risk of future incidence of radiographic knee OA in those without knee OA at baseline did not discover any association between the metabolic equivalent of a task score and the risk of future radiographic knee OA diagnosis (Gates et al., 2022).

Taking a curvilinear approach to analysis, another study discovered an alternative relationship between joint tissue health and physical activity. In 205 middle‐aged men and women without knee OA who were followed for 4 years, cartilage T2 relaxation time mapping (measure of water content and collagen integrity) worsened across all activity levels (Lin et al., 2013). Notably, the magnitude of worsening in various joint compartments was significantly greater in the highest tertile of Physical Activity Scale for the Elderly (PASE) score in comparison to the middle tertile, but the lowest tertile was not significantly different. Further analysis revealed that both the top and bottom 15% of PASE score had significantly greater T2 values in comparison to the middle 70% (Lin et al., 2013). Consequently, those who engage in moderate levels of physical activity might reflect optimal cartilage ageing (natural degeneration of the joint with age), which protects the joint from unhealthy ageing (joint disease) via inactivity.

These contrasting results suggest that a higher physical activity level may both attenuate or induce greater magnitudes of joint degeneration in middle‐aged people or older adults without compromised joint health (e.g., cartilage defects) or knee OA; however, the effects appear to be tissue specific. For example, Xu et al. (2023) highlighted how studies often present either no association or a positive association between physical activity level and cartilage size, but negative associations with the presence of cartilage defects. Interestingly, those who engage in higher levels of weight‐bearing activities but have smaller muscles that act at the knee joint relative to their anthropometrics might be at heightened risk for joint tissue health degeneration, which might have preventative and therapeutic implications.

### 6.2 With and without knee osteoarthritis and osteoarthritic features

A best‐evidence synthesis of 28 studies of heterogeneous designs (case–control, cross‐sectional and longitudinal) in healthy, middle‐aged to elderly men and women both with and without knee OA revealed strong evidence (from multiple high‐quality cohort studies) for a positive relationship between physical activity and osteophytes, no relationship between physical activity and joint space narrowing and a negative relationship between physical activity and cartilage defects (Urquhart et al., 2011). Limited evidence was also presented by a single‐cohort study and two cross‐sectional studies to show a positive relationship between physical activity and cartilage volume (Urquhart et al., 2011). Despite suggestions that physical activity might be beneficial for joint health, several other studies have again suggested a negative impact on joint tissue health. One cross‐sectional study reported a negative association between physical activity and cartilage T1ρ and T2 relaxation times in 151 middle‐aged men and women with and without knee OA (Kumar et al., 2014). Another two studies discovered that higher levels of physical activity in middle‐aged and elderly men and women with compromised joint health at baseline (cartilage defects or bone marrow lesions) did not slow joint deterioration, as noted in people without defects at baseline, and was even associated with accelerated deterioration of the compromised structure (Dore et al., 2013; Teichtahl et al., 2012). Additionally, marathon training and participation have been shown to exacerbate the presence and severity of asymptomatic cartilage defects (Horga et al., 2019). Mechanistic evidence of certain types of activity being deleterious for joint health is provided by a recent study measuring the serum concentrations of metabolites reflecting degradation of the key hyaline cartilage proteins aggrecan and type II collagen, revealing that a lower‐body resistance‐exercise intervention resulted in elevated concentrations of degradation markers at 3 and 6 months, in comparison to baseline (Thudium et al., 2023). Furthermore, the group that engaged in higher‐intensity resistance training, thereby elevating the mechanical load on the joint, presented a greater increase in markers of aggrecan breakdown than those in the lower‐intensity exercise group (Thudium et al., 2023). Similar to those without compromised joint health, the results from these studies suggest higher levels of joint degeneration for middle‐aged people or older adults with baseline knee joint abnormalities or OA who engage in higher levels of physical activity.

Conversely, recent systematic reviews have concluded that exercise interventions are not harmful to joints based on their effect on molecular biomarkers and imaging to quantify the articular cartilage structure and integrity in people at increased risk of, or with, knee OA (Bricca, Juhl et al., 2019; Bricca, Struglics et al., 2019). Bricca, Struglics et al. (2019) using meta‐analysis techniques, discovered no statistical differences between baseline and post‐exercise intervention for a series of pro‐inflammatory cytokines and three cartilage‐specific biomarkers. Taking a narrative synthesis approach within the same review consisting of 57 study comparisons, 63% of studies did not present differences in biomarkers of metabolites, 30% demonstrated a trend for decrease, and 7% demonstrated a trend for increase following exercise. Of note is that despite the exercise interventions employed in the studies included in this meta‐analysis, there was no reduction in common systemic pro‐inflammatory markers, which typically decrease as a consequence of adipose tissue mass reduction (Forsythe et al., 2008). Furthermore, in many of the studies included in the review, body mass or adiposity level was not measured pre‐ and post‐intervention, hence the results regarding inflammation are ambiguous.

In a separate review, Bricca, Juhl et al. (2019) discovered that among those with established knee OA, six studies found that knee joint‐loading exercises had no effect on cartilage thickness, volume or defects. Regarding cartilage composition, one study reported no effect on levels of glycosaminoglycan content, whereas another found a negative effect on glycosaminoglycan content in the medial femoral condyle. Contrarily, the same exercise intervention that showed negative effects on glycosaminoglycan levels demonstrated a positive effect on collagen content in the posterior medial femoral condyle and central medial tibial condyle, assessed through T2 mapping. Additionally, two publications from the same randomized controlled trial reported a positive effect on collagen in the patellar cartilage but no effect on the medial femoral condyle. Lastly, one study found no effect on collagen composition as measured by T2 mapping. Within‐group analyses revealed that all but 1 of 14 studies discovered positive outcomes, such as increased cartilage volume and improved glycosaminoglycan levels (Bricca, Juhl et al., 2019). This suggests that physical activity might have differential effects on protein metabolism within the cartilage, which highlights the complexity of the relationship, given the potential different metabolic responses on key structural and functional proteins within the same tissue. This also suggests that analysis of one marker in isolation as an inference of one aspect of tissue health might not elucidate this complex relationship, and there is a need for the concomitant measurement of several key markers reflecting the metabolism of proteins such as type II collagen and aggrecan.

Bricca, Juhl et al. (2019) and Bricca, Struglics et al. (2019) acknowledge that the overall quality of evidence is low in both reviews. Despite this recognition, the conclusion of both reviews that exercise is not harmful to joints could be interpreted as an example of methodological overreach that compounds the issue of uncertainty. In summary, as demonstrated by these conflicting findings, there is a clear lack of certainty on the effect of physical activity on joint health in those with and without knee OA.

## 7 INFERENCES FROM ACUTE EXERCISE

We can also draw inferences on the impact of physical activity by observing the acute physiological response to joint‐loading exercise. The magnitude of knee joint loading is positively associated with cartilage deformation in a dose–response manner (Eckstein et al., 2005). Intriguingly, greater mechanical load placed on the joint results in a larger volume of fluid effused from cartilage (Heckelman et al., 2020). This perhaps explains why higher joint loading is associated with elevated serum concentrations of cartilage oligomeric matrix protein (COMP) and type II collagen metabolites after weight‐bearing exercise (Dreiner et al., 2020; Roberts et al., 2019), and an increase in serum COMP is correlated with a decrease in synovial fluid COMP (Hyldahl et al., 2016). In 16 participants with knee OA, changes in the serum concentration of a biomarker of aggrecan anabolism (chondroitin sulphate 846 epitope) 5.5 hours after a 30 min walk, expressed as a percentage change relative to the mean of the cohort 30 min after the walk, showed a strong positive correlation with an increase in cartilage thickening of the lateral femur at 5 years follow‐up (Chu et al., 2018). Conversely, the percentage change relative to the mean of the cohort at the 0.5 h time point to 5.5 hours after the walk in a serum biomarker of type I and type II collagen degradation showed a strong negative association with cartilage thinning of the lateral tibia (Chu et al., 2018). In accordance with these findings, the increase in serum COMP concentration in 12 participants without knee OA from 30 minute before the walk to 5.5 hours after the walk showed a strong negative association with loss of anterior medial tibial cartilage (Erhart‐Hledik et al., 2021).

Several blood OA biomarkers fluctuate following exercise (Bjerre‐Bastos et al., 2023, 2024). However, a clear elucidation of the impact of exercise modality and intensity is lacking. Furthermore, there is a paucity of information on the time course of biomarker response post‐exercise. Serum COMP concentrations are widely reported to increase 30 minute after cessation of walking and decrease to baseline resting levels by 60 minute post‐exercise, and this increase has been linked to future development of knee OA (Roberts et al., 2019). However, there is a possibility that the initial post‐exercise increase is a consequence of load‐inducing effusion of the synovial fluid. Those experiencing heightened disease activity might have elevated concentrations of degradation markers in the synovial fluid, and when a shift of this fluid to the circulating blood occurs via mechanical compression, those with higher catabolic activity in the joint may present higher concentrations of degradation metabolites in the blood. Intriguingly, an increase in serum COMP after a 30 minute walk has been observed at 5 hours post‐exercise, which might, in turn, reflect catabolic activity, rather than removal of metabolites from the synovial fluid (Mündermann et al., 2005). Considering that joint tissues have a far greater protein turnover than previously suggested and at similar rates to skeletal muscle (Smeets et al., 2019), a highly plastic tissue, further investigation of the time course of OA biomarker fluctuations in response to joint loading is required.

Interestingly, a higher body mass index and body fat percentage are associated with greater deformation and higher T1ρ scores, indicating a lower proteoglycan content (Collins et al., 2018). However, physical activity level does not appear to alter the morphology of the cartilage in response to loading (Eckstein et al., 2005; Heckelman et al., 2020). This suggests that for individuals with obesity, it is crucial to choose the right type of physical activity to avoid excessive joint loading that could lead to cartilage damage. This could consist of alternative forms of exercise modalities that do not involve repetitive high force transfer at the knee joint in those with a high body mass, given that non‐weight‐bearing activities to improve aerobic fitness and increase energy expenditure would still induce beneficial results in terms of knee OA risk by ameliorating chronic inflammation. In those with knee OA, greater amounts of knee articular cartilage deformation in response to loading has been observed than in those with healthy cartilage (Cotofana et al., 2011). This is complemented by evidence that greater changes in femoral and tibial cartilage relaxation times in response to loading were present in the knee OA group in comparison to control subjects, suggesting a higher level of fluid effusion, which suggests impaired dissipation of loading (Souza et al., 2014). In addition to morphological and structural changes, a differential systemic biomarker response to weight‐bearing physical activity has been observed between individuals with knee OA and those without (Roberts et al., 2019). This is potentially explained by individuals with knee OA presenting with higher resting levels of metabolites related to cartilage degradation in comparison to those without knee OA (Hao et al., 2019). Consequently, people with knee OA might exhibit an attenuated increase in serum COMP in response to physical activity in comparison to those without knee OA, indicating that knee OA affects the biochemical response to mechanical stress on the joints. When combining these inferences, perhaps those at most risk of joint maladaptation to loading are those who are already experiencing knee OA and are living with obesity.

## 8 WHAT DOES THIS MEAN?

Considering the information garnered from in vitro and ex vivo research, animal models and tissue responses to exercise, several inferences can be drawn about physical activity and knee OA. In vitro and ex vivo studies suggest that moderate mechanical loading can stimulate beneficial changes in joint tissues, such as increased anabolism and attenuated catabolism of key structural and functional proteins and reduced inflammation. Animal models support this, indicating that moderate levels of physical activity can minimize tissue degradation, whereas both low and high levels of activity can lead to joint damage. Acute exercise responses also illustrate that although mechanical loading can increase markers of cartilage stress, appropriate types and amounts of physical activity might not necessarily harm joint morphology.

In those with obesity, responses to joint‐loading exercise might be different from the responses in those with a lower level of adiposity, with the cartilage potentially being more susceptible to overloading, thereby generating pathophysiological perturbations of the mechanosensitive joint tissues. Another body composition factor that might influence knee OA disease pathogenesis is reduced lower‐body lean mass index, whereby reduced lower‐body strength reduces shock absorption and promotes instability. A sedentary lifestyle, whereby the adiposity level is high and lower‐body muscle mass low, could be highly deleterious for joint health, which has been postulated to be a key reason why the rates of knee OA have doubled since the pre‐industrial era (Wallace et al., 2017). This perhaps suggests that the transition from a sedentary lifestyle to a more physically active lifestyle is crucial to facilitate joint health, but it must be managed carefully.

**FIGURE 1:**
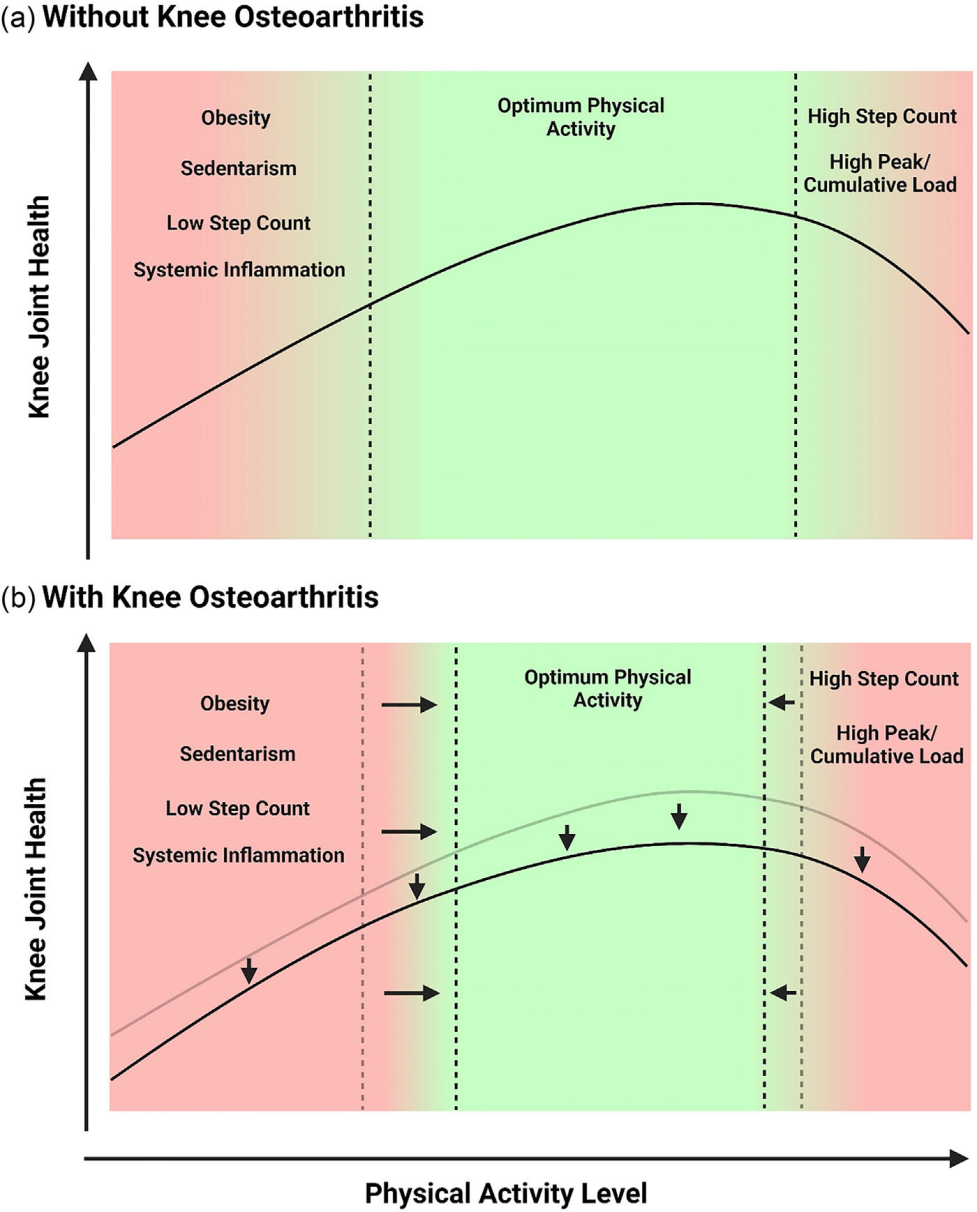
Hypothesized associations between physical activity, mediators such as obesity, sedentary behaviours and knee joint health in those without and with knee osteoarthritis.

There might potentially also be differential responses between those with knee OA and those without. Indications exist that individuals with OA might have a reduced capacity to sustain higher magnitudes or repetitions of loading. Collectively, these findings suggest an optimal zone of activity that balances joint health benefits without causing harm. Yet for those with knee OA, the capacity of the joint to sustain underloading and higher loading might be different in comparison to those without the condition (Figure 1). At present, this is theoretical. Further research is required to understand the relationships of the type of activity and the physiological response to mechanical loading with joint tissue metabolism to help develop physical activity guidelines for those at risk of knee OA and those currently experiencing the condition.

Given the contradicting data from human studies, caution is still required when drawing these inferences. The discrepancies in human studies highlight the complexity of the relationship between physical activity and knee OA. However, these discrepancies might arise from methodological flaws. Addressing these sources of contradiction, uncertainty and ambiguity might help to untangle the complexities and provide clearer guidelines for managing knee OA through physical activity.

## 9 SOURCES OF UNCERTAINTY AND AMBIGUITY

Sources of uncertainty and ambiguity that contribute to the often‐contrasting evidence base need to be identified and discussed to contextualize previous findings and guide future studies on overcoming methodological limitations.

### 9.1 Incommensurability and subjective questionnaires

Most studies investigating knee OA use subjective questionnaires to measure physical activity levels. Although convenient and resource efficient, these questionnaires fail to quantify accurately key variables that directly impact knee OA, such as energy expenditure and cumulative joint load. This limitation exemplifies the concept of incommensurability, whereby certain quantities cannot be compared directly or measured in a meaningful way. Supporting this notion is that subjective physical activity measurements are used to infer energy expenditure, although the accuracy is limited. Even the most validated and calibrated self‐report questionnaire exhibits significant limitations in estimating energy expenditure precisely (Pearce et al., 2020). Subjective physical activity is also used to infer the mechanical load of an individual. A higher physical activity level is often used crudely to suggest a higher level of mechanical load on the knee joint. Efforts have been made to categorize physical activities into having different mechanical loading magnitudes by calculating a mechanical component score attributed to a ground reaction force magnitude that has been normalized to body mass (e.g., walking = 1, running = 3) (Verweij et al., 2010). Although this can help to distinguish activities that expose the knee joint to higher levels of force transfer, the relationship between ground reaction forces and internal tissue loads is poorly correlated even during repetitive movements, such as running (Matijevich et al., 2019). Consequently, subjective physical activity questionnaires that provide an index of mechanical load can be interpreted as a simplification and might potentially not provide data of a quality required to deduct meaningful inferences.

### 9.2 Missing mechanical link

Knee OA is influenced by both physiological and mechanical factors through mechanotransduction, which is the process by which cells sense and respond to mechanical stimuli, such as perturbations, and which plays a crucial role in joint tissue metabolism (Vincent & Wann, 2019). In vivo, the mechanical stimulus can be estimated by measuring biomechanics. However, the majority of research does not consider the impact of biomechanics during weight‐bearing physical activities. This limits our understanding of the relationship between knee OA and physical activity, considering that biomechanics during locomotion influence the magnitude of mechanical loading at the knee. An example of this is that during walking, acceleration increases the magnitude of knee joint loading, and running increases loading further (Miller, 2017).

In vivo research often relies on inferences from a single laboratory session to study the relationship between mechanical loading and knee OA or to predict future joint degeneration. This approach assumes consistent gait patterns, overlooking both the variability in human gait and the frequency of contacts a joint is exposed to during daily living. The term ‘cumulative joint load’ refers to the total load experienced by the joint during daily activities, considering the magnitude of force, frequency (step count) and duration (time of stance phase/loading) (Maly, 2008). Physical activity levels are often quantified using step counts per day, and although this provides some indication of time spent in physical activity and the quantity of single joint compressions, it does not offer insights into the magnitude or duration of the load. Maly (2008) proposed a method combining knee adduction moment (KAM) with step count to estimate cumulative joint loading, whereby the mean KAM impulse from a single laboratory session is multiplied by the number of steps per day outside of the laboratory. Although valuable, this approach misses important data about the temporal and magnitude aspects of loading, because a pedometer treats all steps equally, regardless of walking or running. However, KAM peak and impulse, rather than loading frequency, are associated with cartilage thickness in the medial tibia and femur in knee OA patients (Maly et al., 2015). Moreover, research indicates that larger KAM peaks and impulses, especially when interacting with body mass index, predict cartilage volume loss in the medial tibia over 2.5 years in individuals with knee OA, whereas cumulative load incorporating step count does not (Brisson et al., 2017). This perhaps implies that peak forces applied to the joint provide a more salient mechanical load variable in the context of knee OA disease. Despite the shorter ground contact duration during running, cumulative joint loading is similar to walking but with higher peak forces (Miller, 2017). Further complicating the relationship of mechanical loading and tissue degeneration is the status of tissue pathology. The relationship between joint loading and metabolism might potentially differ between individuals with and without knee OA, and the sum of contact force peaks might be more critical than cumulative load using impulse.

Further mechanical understanding needs to be developed to improve our understanding of joint loading, because there is a paucity of data using internal loading parameters. There are long‐standing misconceptions of ground reaction forces being indicative of joint loading, which has been disproved empirically (Matijevich et al., 2019). Although KAM is commonly used as a surrogate for total and medial reaction or contact forces, it quantifies external loads rather than internal joint mechanics. Improved understanding of the relationship between mechanical loading and physical activities, such as walking, can be achieved through the adoption of musculoskeletal modelling techniques, which can quantify the internal loading of the joints more accurately; for example, joint reaction forces, which incorporate muscle activation dynamics, to provide a more comprehensive understanding of internal joint loading, with external knee moments explaining the variance in joint reaction force measurements only in part (Holder et al., 2020).

In addition to incorporation of musculoskeletal modelling to enhance specificity of mechanical loading, advances in wearable technology and signal‐processing methodology now demonstrate promising progression towards the accurate estimation of spatiotemporal parameters (Bergamini et al., 2012), joint kinematics (Mundt et al., 2020), ground reaction forces (Carter et al., 2024a) and surrogates of knee joint loading outside of the constrained laboratory environments (Carter et al., 2024b). This progress better enables studies to explore the intricate relationships between physical activity, biomechanics and knee OA. However, owing to the relative novelty of these approaches, widespread adoption is limited, and the lack of routine methodological approaches hinders our understanding of the connection between physical activity and knee OA. Furthermore, despite the link between higher mechanical loads and knee OA, and the potential for knee load modification through gait retraining, approved/recommended biomechanical interventions to slow joint degeneration do not exist.

### 9.3 Oversimplistic univariate modelling approaches

**FIGURE 2:**
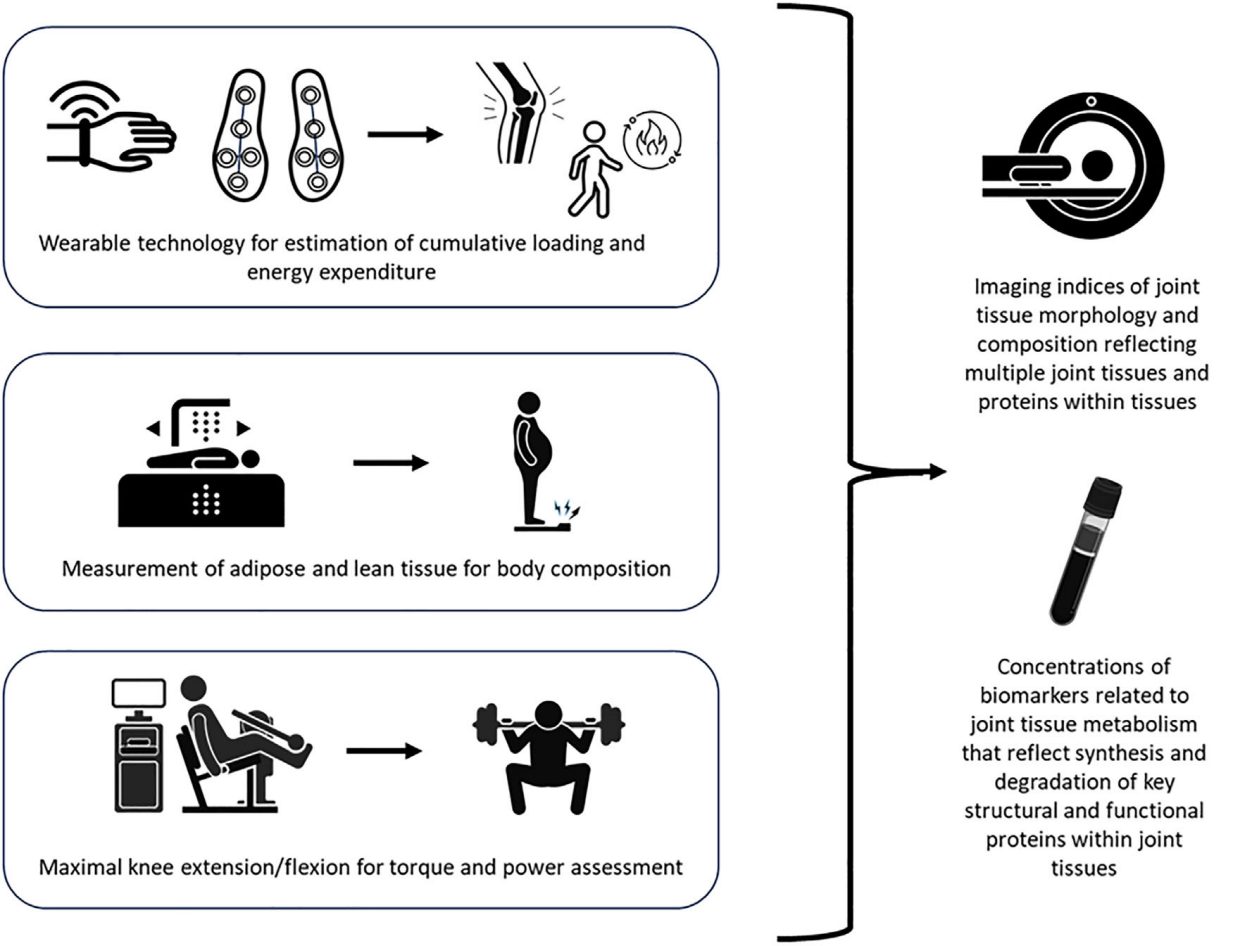
Variables responsive to exercise interventions that might influence joint tissue health and need to be measured during exercise interventions or during observation to help understand the association between physical activity and joint health.

Univariate models might oversimplify the complex relationship between physical activity and knee OA by not accounting for the interactions between multiple influencing factors. To address this complexity, multivariate analysis is required and would provide a more nuanced understanding. For example, energy expenditure, mechanical load and the level of adiposity are all related to the physical activity level of an individual and exert influence on joint tissue metabolism. Multivariate analysis could therefore help to establish whether observed joint deterioration can be explained by energy expenditure, mechanical loading or higher body fat percentages among participants. Furthermore, when designing randomized controlled trials, monitoring the exposures appropriately is essential to understanding the effect of the exercise intervention. For example, based on the exercise intervention, is it the increased cumulative joint loading, the reduction in adipose tissue mass, increased energy expenditure or the increased muscle size that is inducing the physiological change, be it positive or negative, in the joint tissues? Consequently, monitoring or controlling these variables is essential in the design of studies investigating the effects of physical activity on joint health (Figure 2).

### 9.4 Non‐linear relationships

These contrasting results might be attributable to the limitations of linear analysis approaches. There is a paucity of research investigating the relationship between physical activity and knee OA disease, with only Lin et al. (2013) adopting a non‐linear statistical approach for human in vivo research. The relationship between physical activity and knee OA might be non‐linear, meaning that both very low and very high levels of activity could be detrimental, whereas moderate levels might be protective. Curvilinear analysis can identify these non‐linear patterns, offering insights that linear models regularly used in the field might overlook.

## 10 CONCLUSION

Although the benefit of engaging in regular bouts of physical activity on knee OA illness is effective and efficacious, the effects of exercise on knee OA disease remain ambiguous. Although in vitro and ex vivo research and in vivo animal models suggest that moderate physical activity can promote knee joint tissue health through mechanotransduction, maintaining homeostasis and reducing the impact of adiposity‐driven chronic inflammation, human studies provide less definitive conclusions. The reliance on self‐reported physical activity data in human research introduces significant limitations owing to lack of specificity and potential biases, resulting in contrasting and confounding findings. To advance our understanding, future research must adopt more precise and objective measures of physical activity to ascertain its physiological and mechanical impacts on joint health. These objective measures should quantify energy expenditure, mechanical loading, adiposity and muscle strength to gain a better understanding of the relationship between physical activity and knee OA. Until such methods are used appropriately, the effects of physical activity on knee OA pathogenesis will continue to be unclear, impacting the ability to provide evidence‐based guidelines for exercise recommendations and potentially leading to suboptimal management of knee OA in clinical practice.

## AUTHOR CONTRIBUTIONS

CRediT: Karl Morgan: conceptualization, investigation, writing—original draft. Joshua Carter: writing—review and editing. Dario Cazzola: writing—review and editing, supervision. Jean‐Philippe Walhin: writing—review and editing, supervision. All authors approved the final version of the manuscript and agree to be accountable for all aspects of the work in ensuring that questions related to the accuracy or integrity of any part of the work are appropriately investigated and resolved. All persons designated as authors qualify for authorship, and all those who qualify for authorship are listed.

## CONFLICT OF INTEREST

The authors declare that they have no conflicts of interest.
